# Dental professionals’ experiences of managing children with carious lesions in their primary teeth – a qualitative study within the FiCTION randomised controlled trial

**DOI:** 10.1186/s12903-020-1051-7

**Published:** 2020-03-04

**Authors:** Zoe Marshman, Jennifer E. Kettle, Richard D. Holmes, Kathryn B. Cunningham, Ruth Freeman, Barry J. Gibson, Elaine McColl, Anne Maguire, Gail V. A. Douglas, Janet E. Clarkson, Nicola P. T. Innes

**Affiliations:** 1 School of Clinical Dentistry, Claremont Crescent, Sheffield, S10 2TA UK; 2 School of Dental Sciences, Faculty of Medical Sciences, Newcastle University, Framlington Place, Newcastle upon Tyne, NE2 4HH UK; 3 School of Medicine, University of St Andrews, North Haugh, St Andrews, KY16 9TF UK; 4 Dental Health Services Research Unit, School of Dentistry, Park Place, Dundee, DD1 4HN UK; 5 Newcastle Clinical Trials Unit, 4th Floor, William Leech Building, Framlington Place, Newcastle Upon Tyne, NE2 4HH UK; 6 Leeds Dental Institute, Clarendon Way, Leeds, LS2 9LU UK; 7 School of Dentistry, University of Dundee, Park Place, Dundee, DD1 4HN UK

**Keywords:** Dental caries, Carious lesions, Paediatric dentistry, Primary care, Randomised controlled trial, Qualitative research, Dentists, Dental professionals

## Abstract

**Background:**

The lack of evidence for the effective management of carious lesions in children’s primary teeth has caused uncertainty for the dental profession and patients. Possible approaches include conventional and biological management alongside best practice prevention, and best practice prevention alone. The FiCTION trial assessed the effectiveness of these options, and included a qualitative study exploring dental professionals’ (DPs) experiences of delivering the different treatment arms. This paper reports on how DPs managed children with carious lesions within FiCTION and how this related to their everyday experiences of doing dentistry.

**Methods:**

Overall, 31 DPs from FiCTION-trained dental surgeries in four regions of the UK participated in semi-structured interviews about their experiences of the three treatment arms (conventional management of carious lesions and prevention (C + P), biological management of carious lesions and prevention (B + P) or prevention alone (PA)). A theoretical framework, drawing on social practice theory (SPT), was developed for analysis.

**Results:**

Participants discussed perceived effectiveness of, and familiarity with, the three techniques. The C + P arm was familiar, but some participants questioned the effectiveness of conventional restorations. Attitudes towards the B + P arm varied in terms of familiarity, but once DPs were introduced to the techniques, this was seen as effective. While prevention was familiar, PA was described as ineffective. DPs manage children with carious lesions day-to-day, drawing on previous experience and knowledge of the child to provide what they view as the most appropriate treatment in the best interests of each child. Randomisation undermined these normal choices. Several DPs reported deviating from the trial arms in order to treat a patient in a particular way. Participants valued evidence-based dentistry, and expect to use the results of FiCTION to inform future practice. They anticipate continuing to use the full range of treatment options, and to personally select appropriate strategies for individual children.

**Conclusions:**

RCTs take place in the context of day-to-day practices of doing dentistry. DPs employ experiential and interpersonal knowledge to act in the best interests of their patients. Randomisation within a clinical trial can present a source of tension for DPs, which has implications for assuring individual equipoise in future trials.

## Background

The majority of dental care for children in the UK is provided directly by primary care general dental practitioners (GDPs) and their teams. However, research has provoked debate around effective management of carious lesions in primary teeth after three studies which were carried out in primary care indicated that the clinical outcomes following removing carious tissue and restoring primary teeth are not significantly different from leaving teeth unfilled [1–3].

Current clinical guidelines for managing carious lesions in young children produced by the British Society of Paediatric Dentistry are largely based on evidence from studies conducted in secondary care (i.e. hospital settings) or specialist paediatric practice [4, 5]. These guidelines are not always seen as applicable to primary care and are not always followed in general dental practice [6].

This lack of evidence on effective and efficient management of carious lesions in children’s primary teeth, when treated in primary care, continues to cause uncertainty for the dental profession as well as parents and children. In view of insufficient evidence on which to base a recommendation as to which carious lesion management strategy is most effective within primary care, a multi-centre, three-arm, parallel group, patient-randomised controlled trial (RCT) was undertaken to address this deficiency [7].

### The FiCTION trial

The Filling Children’s Teeth: Indicated Or Not (FiCTION) RCT was designed with the primary objective of comparing the incidence of dental pain and dental infection experienced over a period of 3 years in 3–7 year-old children with carious lesions in primary teeth when managed by one of three treatment strategies (hereafter referred to as the trial arms) [7]. The arms were multi-component interventions as follows:

- Best practice prevention alone (PA). This involved four components or pillars, carried out according to current national guidelines: toothbrushing/self-applied topical fluoride use; dietary investigation, analysis and intervention; fissure sealants applied to permanent teeth; fluoride varnish applied to primary and permanent teeth.
- The conventional management of carious lesions, with best practice prevention (C + P). Local anaesthetic (LA) was administered, carious tissue was mechanically removed and a restoration was placed to restore the cavity. Best practice prevention was carried out as above.
- The biological management of carious lesions, with best practice prevention (B + P). Carious tissue was sealed into the tooth, and separated from the oral cavity by application of an adhesive restoration material over the carious tissue, or by covering with a PMC. Best practice prevention was carried out as above.

Children with at least one primary molar tooth with a carious lesion involving dentine were randomly allocated to one of the three treatment arms [7] and their carious lesion(s) managed, according to the treatment arm to which the child was randomised, for up to 3 years.^1^ All treatment was recorded by Dental Professionals (DPs), including any treatment delivered outside the allocated arm. The trial found that there was no evidence of difference among the three treatment approaches for incidence or number of episodes of dental pain and/or infection [8]. A report on the secondary outcomes is in press [9].

### Qualitative research in clinical trials

Qualitative research can be used within clinical trials to help optimise interventions; improve the design, conduct and process of trials; consider variation in outcomes; determine the accuracy of measures, and; understand participant experiences of the target condition [10]. A fundamental premise of RCTs is clinical equipoise; that is, there is insufficient evidence to state that one intervention is better than another [11]. Qualitative research has also addressed how the need to be in (individual) equipoise poses a challenge for healthcare professionals involved in RCTs [12]. The results of RCTs can be difficult to apply to routine care, unless the target behaviour is explored within its everyday social context [13].

This is important because clinical trials represent only part of what DPs do on a day-to-day basis, and thus the activities involved are situated within the wider context of ‘doing dentistry’. In this article, we use the phrase ‘doing dentistry’ to refer to the everyday work of DPs including dentists, dental therapists and hygienists, dental nurses and practice managers. In order to apply the lessons of clinical trials to routine healthcare, it is necessary to recognise how trial- and clinical-protocols relate to healthcare professionals’ prior experiences, everyday activities and ways of thinking about the issue under investigation.

One way of theorising what happens in clinical trials is through Social Practice Theory (SPT). SPT seeks to explain how human activities are organised across individuals, how the standards of such activities are set and recognised, and how these activities develop and change over time [14–17]. SPT can help to situate activities that are the target of a trial within the wider context of what DPs do on a daily basis (‘doing dentistry’). This can help highlight where resistance to change may arise and why. It is also a useful point of overlap between public health and social science [18, 19] and has been used to theorise ‘unhealthy’ practices including drinking, eating and smoking [20–22].

Within hospitals, healthcare professionals have been observed to enact a range of social practices, which combine and coordinate to deliver care [23]. Similarly, the dental surgery is a setting for multiple social practices that constitute ‘doing dentistry’. Doing dentistry involves managing patients with carious lesions, as well as various other activities, such as managing patients with other oral diseases, gaining patient consent, infection control and team-working. What dentists do can change as a result of participating in research, which can involve unfamiliar practices, or familiar practices carried out in different circumstances. Using SPT as a theoretical framework helps us study how social change happens and this is why it was useful in this study.

A qualitative study was integrated into FiCTION to explore the perspectives of clinicians and participants. The aim of the qualitative research with DPs was to:

- Explore the experiences of DPs in providing the three treatment strategies
- Explore whether previous experience has an impact on their preferences
- Identify training needs in delivering the treatment strategies
- Explore how experiences of the trial will shape how DPs manage children with carious lesions in the future [24].

This paper uses SPT to illustrate primary care DPs’ experiences of managing children with carious lesions within this randomised controlled trial, and relates these to their experiences of primary care dentistry more generally.

## Methods

Qualitative interviews were used to explore DPs’ experiences of providing the three treatment arms within the FiCTION trial. The study was approved by the Health Research Authority East of Scotland Research Ethics Service (12/ES/0047). Local Research and Development approval was also provided by the relevant NHS Trust or Health Board for each participating dental surgery. All participants provided written informed consent.

### Participants

Participants were DPs selected from the list of 68 dental surgeries participating in the trial in the four regions where the qualitative sub-study was taking place: Scotland, North East England, Yorkshire and London. Participants were identified by means of purposive maximum variation sampling using the variables of gender, time since qualifying, number of FiCTION child participants at their dental surgery, research experience, dental setting (community/public dental service or general dental service) and regional location [25]. The sample also included those who had recorded instances of having deviated from the FiCTION clinical protocol for a variety of reasons, as these were cases of particular interest.

### Semi-structured interviews

Participants were offered a choice of individual or group interviews with other members of the dental team and informed that the aim of the qualitative research was to explore their views of the three treatment arms and how well they worked in primary care. Individual interviews were carried out either in-person or by telephone, while group interviews took place in dental surgery premises or at the universities involved in the research. Interviews were carried out by four researchers, all with previous experience of conducting qualitative interviews. Interviews continued until no new data emerged and were audio-recorded, transcribed by an external company and checked for accuracy, with corrections made where necessary. The data were anonymised by the use of study numbers and omission of any identifying information during transcription.

The interviews followed a topic guide derived from the literature on behaviour change [14, 26], process evaluation [27, 28] and the implementation of research findings in clinical practice [29, 30] and discussions with the trial management group (see Additional file 1 for the topic guide). This enabled a thorough exploration of the DP’s perspectives on carrying out the three treatment arms, how the three arms were delivered in practice and the contextual factors that influenced this.

### Data analysis

**Fig. 1:**
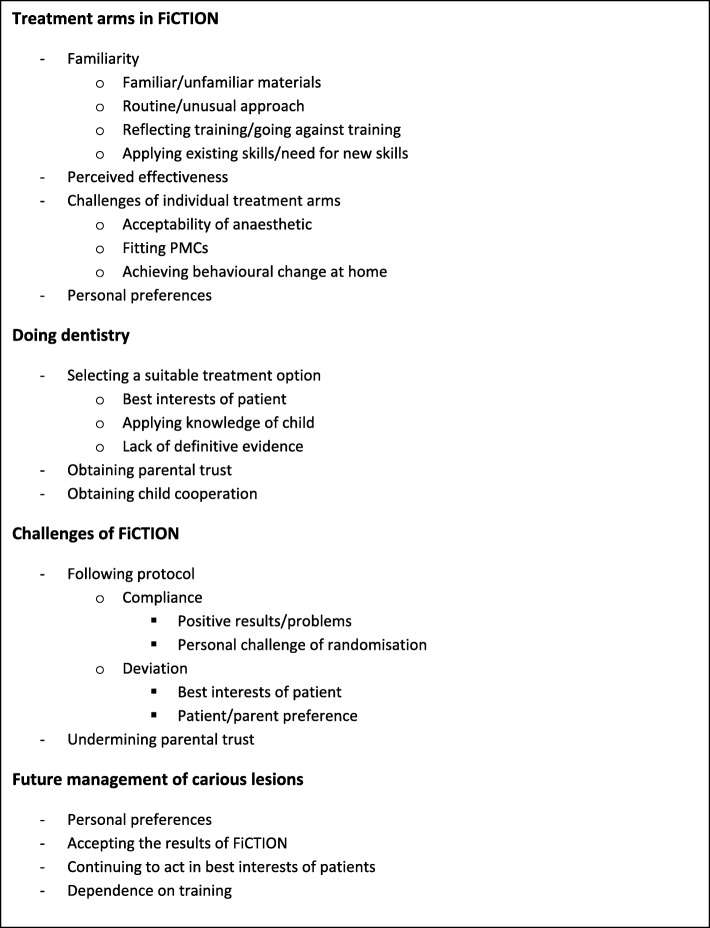
Thematic framework

Interview transcripts were imported into the qualitative data analysis software NVivo 11 (QSR International, Warrington, UK)™ for coding and management. The data were analysed using Framework Analysis [31] and the results theorised using SPT. The data were primarily analysed by two researchers with experience of qualitative research on various topics related to oral health and dentistry (JK and ZM), one of whom (ZM) is dentally qualified. Both read and re-read all the transcripts to achieve familiarisation and identified recurring ideas or themes for discussion with BG, RH and NI. A conceptual framework was then devised based on the emerging themes and framework-based codes were applied to all the data by JK. The framework was refined through the coding process as additional themes emerged from other transcripts (see Fig. 1 for the final framework). Coding was checked in 10 % of the transcripts (*n* = 3) by a second member of the research team (ZM).

## Results

Overall, 31 DPs were interviewed (see Table 1 for participant characteristics).

**Table 1:** Dental professional participant characteristics

Code	Professional role	Community or general dental service	No. patients recruited	Research experience (prior experience of dental research)	Time (y) since qualifying	FiCTION region
D01	Dentist	Community	17	Yes	37	Scotland
D02	Dentist	General	24	Yes	30	Scotland
D03	Dentist	Community	4	No	17	Scotland
D04	Dentist	General	23	No	20	Scotland
D05	Dentist	General	36	Yes	18	Scotland
E01	Dentist	General	2	No	10	Scotland
G01	Dentist	General	24	Yes	20	Scotland
L01	Dentist	Community	22	Yes	14	Yorkshire
L02	Dental Nurse	Community	22	No	25+	Yorkshire
LDN01	Dentist	General	37	No	27	London
N01	Dentist	General	20	No	17	North East
N02	Dentist	General	14	Yes	16	North East
N03	Dentist	General	14	No	2	North East
N04	Senior Dental Nurse	General	30	No	15	North East
N05	Practice Manager	General	24	No	13	North East
N06	Dentist	General	30	No	36	North East
N07	Dentist	General	30	Yes	16	North East
N08	Dentist	General	15	No	7	North East
N09	Dental Therapist	General	17	No	4	North East
N10	Dentist	General	17	No	17	North East
N11	Dental Therapist	General	17	No	2	North East
N12	Dentist	General	17	No	10	North East
N13	Dental Nurse	General	17	Yes	8	North East
S01	Dentist	General	4	No	16	Yorkshire
S02	Dental Nurse	General	4	No	Not given	Yorkshire
S03	Practice Manager	General	4	No	N/A	Yorkshire
S04	Dentist	Community	22	Yes	14	Yorkshire
S05	Dentist	General	21	No	11	Yorkshire
S06	Dentist	General	21	No	8	Yorkshire
S07	Dentist	General	32	Yes	15	Yorkshire
S08	Practice Manager	General	32	No	N/A	Yorkshire

DPs described their experiences of the three treatment arms (conventional and prevention, biological and prevention, and prevention alone), and how these related to everyday practice. The first section of the results demonstrates how each of the treatment arms can be understood as social practices, comprising ‘entities’ that are repeatedly performed both within the trial and in day-to-day work. The second section explores how these practices are ‘bundled together’ as ‘managing children with carious lesions’, both within and outside of the trial. These findings are illustrated with quotations from the interviews.

This paper uses terminology from SPT. Within SPT, practices can be analysed as ‘entities’ (that is, a recognisable activity that can be spoken about, written about, taught etc.) and ‘performances’ (the repeated doing of this activity) [15]. ‘Practices-as-entities’ refers to recognisable configurations of interconnected elements; particular materials (tangible physical things, technologies and infrastructure), competences (understanding and skill) and meanings (symbolic meanings and ideas) are linked together.^2^ The entity is a recognisable *idea* of something; it exists whether or not it is being done at any particular moment. However, in order for it to have that status it needs to be repeatedly performed by different people over time. This means people are ‘recruited’ into social practices; they are introduced to particular entities and initiated into performing these [14]. For DPs, this can happen at dental school, or through continuing professional development. People who are recruited into a social practice repeatedly perform the entity involved, and it is these repeated performances that maintain the practice over time, and reinforce the entity as something recognisable that *can* be performed.^3^

**Fig. 2:**
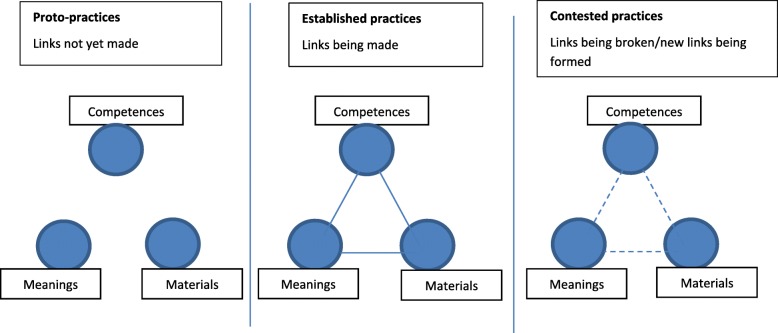
Proto-practices, established practices and contested practices (adapted from Shove et al., 2012 [14])

Initially entities are proto-practices; the elements exist but the links between them are not widely recognised. As the entity is performed repeatedly by different people, in this case DPs, the links are strengthened and a proto-practice can become ‘established’. People can also ‘contest’ a practice by rejecting elements and challenging the links between meanings, materials and competences involved; over time this can weaken the links within an entity and introduce different elements (see Fig. 2). Elements can be superseded, as new technologies and skills are developed, while meanings can be introduced by other people connected to the practice [23].^4^

### Experiences of the treatment arms

The DPs explained their experiences of delivering the three treatment arms. These experiences were mapped according to the theoretical framework of SPT (Table 2).

**Table 2:** DPs’ descriptions of the FiCTION treatment arms

Treatment arm	Materials (tangible physical things, technologies, infrastructure, the stuff of which objects are made)	Competences (understanding, skill, know-how and technique)	Existing Meanings (symbolic meanings, ideas and aspirations already linked into an entity)	Potential Meanings (symbolic meanings, ideas and aspirations that have the potential to be linked into an entity)
Conventional (C + P)	Drill Filling material LA Needle	Removing carious tooth tissue Filling carious lesions Negotiating use of LA with child Injecting LA	‘Familiar’ ‘Supposed to do’ ‘Routine’ ‘Effective’ ‘Off-putting’	‘Ineffective’
Biological (B + P)	PMCs Glass ionomer cement	Identifying the correct size crown Applying the PMC Applying glass ionomer cement	‘Unfamiliar’	‘Familiar’ ‘Effective’
Prevention alone (PA)	Fluoride varnish Fissure sealant Diet sheets	Applying fluoride varnish Providing advice to parents Providing advice to children Achieving behaviour change at home	‘Familiar’ ‘Routine’ ‘Insufficient’	

#### Conventional arm

In the interviews, the DP participants described the conventional arm as what they had ‘always’ or ‘generally’ done, or what they were doing ‘before’:

‘*That conventional arm, that's how we generally treat people anyway. So that's no different to us*.’ (S02: Dental nurse, Yorkshire)

As they were used to providing restorations, some DPs felt this entity had a meaning in terms of what they were ‘supposed to do’ (that is, this was one of the ‘elements’ referred to in SPT) (Table 2):

‘*So, that is a little bit of a difference to get your head around when you maybe see a cavity and you’re not doing anything per se, you’re not picking up a drill, which kind of goes against all of the teaching that you’ve had before.*’ (N08: Dentist, North East)

While the actions of delivering LA, removing carious tissue and restoring a cavity were regarded as ‘conventional’ and effective by some participants, others questioned this approach and its effectiveness:

‘*I think, for me, because I'm accustomed to doing conventional fillings and I'm good at them … ..the fillings would have lasted*.’ (D01: Dentist, Scotland)

‘*I think parents already know and perhaps dentists already know as well, it's very, very difficult to predict what's going to happen to these baby teeth. And whether actually putting an amalgam filling makes any difference whatsoever.*’ (D02: Dentist, Scotland)

This is an example of the importance of considering the performance of a particular entity (for example, the entity of drilling and filling). The way in which this entity is performed can vary, as DPs may have more or less experience, which may impact on the outcome. Here D01 suggests that he/she is particularly skilled in performing this entity (and thus he/she will have a positive outcome). D02 contests the practice, suggesting that outcomes may be variable, regardless of skill. As he/she shows, practices can also be contested by other people involved, such as parents. Contesting a practice can weaken the connection between elements (i.e. the material of filling, the competence of applying it and the meaning of this as an ‘effective treatment for managing carious lesions’).

Negotiating the use of LA was another skill involved in this treatment arm (although some DPs reported removing carious tooth tissue without using LA). The following examples demonstrate how giving LA can be a contested practice among parents and children:

‘*I mean if you can get away with doing local without the kids realising, which you can, if you got a good parent. But if they [the parents] are saying, “Oh, they don’t want the needle. You’re not going to give them the needle.” That’s when you get a problem*.’ (S06: Dentist, Yorkshire)

‘*When it comes to actual anaesthetic with a needle, you find a lot of children at that point is when they stop the treatment [ … ] One of the reasons why I don't often do a conventional way of doing fillings, because of my experience of extractions.*’ (N03: Dentist, North East)

The first quote illustrates the contested nature of LA, which can have a meaning of being ‘off-putting’ to patients. The performance of LA administration requires the skill of negotiating co-operation with the child’s parent. The second suggests that the needle itself is ‘off-putting’, indicating the importance of the materials involved in each practice and how they can carry meaning and generate resistance. Performing the administration of LA generates tension and involves persuading children to accept LA. As a result, difficulties with this aspect of the treatment arm can result in negative connotations being associated with the removal of carious tooth tissue and the subsequent filling of cavities. Repeated performances of the entity of drilling and restoring in which LA is contested can change the entity; the materials and competences involved can take on different meanings (ineffectiveness).

#### Biological arm

While removing carious tooth tissue and restoring teeth was labelled and understood as ‘conventional’ (albeit seen as ‘ineffective’ in some cases), sealing carious tissue into teeth had different meanings. Firstly, the PMCs placed using the Hall Technique were viewed as a particularly effective component of this arm by some DPs:

‘*In terms of the Hall crowns that we have placed in practice, they have lasted surprisingly well. And the patients have been symptom-free*.’ (N01: Dentist, North East)

The use of ‘surprisingly’ here indicates that the meaning of sealing-in of carious tooth tissue is contentious and potentially unstable; importantly, there is scope for the meaning to stabilise as entities evolve through repeated (successful) performance. When participants had prior experience of using the techniques included in the biological arm, and these were part of how they usually treated children with carious lesions, they were generally positive about this approach. For example, dental professionals in Scotland who were accustomed to performing the entity of sealing-in carious tissue with PMCs felt ‘comfortable’, as this entity has settled down and become more acceptable over time:

‘*The biological arm is going great. It’s good because we’re able to add into that, obviously, what my … what I’m more comfortable doing*.’ (D03: Dentist, Scotland)

As well as being a treatment that is understood as effective, the biological approach requires particular competences that can be developed through training (for example, the technique of fitting the PMC).

In terms of their experiences during the trial, participants identified the importance of finding the right size of crown and the amount of time this can take:

‘*I faff around too much, and I’m not really sure of the size and I have to try quite a few on*.’ (S05: Dentist, Yorkshire)

Here, the materials involved (the PMC and the storage box) reinforced the view that performing this entity was not necessarily familiar to all the participants:

‘*Historically there weren’t that many people doing Hall crowns regularly in this area*.’ (N06: Dentist, North East)

The contrast between the ‘conventional’ treatment and the ‘unfamiliar’ entity of sealing-in carious tooth tissue, supports the former as a more established approach. Nevertheless, the DPs in this study demonstrated that increasing knowledge of different treatment options affected how these were understood (particularly in terms of effectiveness), and thus illustrated the potential for the meanings of these activities to change over time. These data suggest ease of performance as being central to the adoption of an activity and demonstrate how familiarity with a practice remains a crucial dimension to the performance of that practice.

#### Prevention alone arm

Participants spoke about prevention activities as part of what they ‘normally’ did. The following quotations reveal how prevention was dependent on patients themselves (and their parents) performing the relevant practices competently. In the following section it will become apparent how this latter point is critical to how DPs saw the performance of the PA arm.

‘*If they had a carious lesion then we would apply fluoride varnish. We'd stress more and more about the oral hygiene and about diet. We would get them in and out more often to see if they were following our advice about fluoride and helping to arrest the caries early before it leads to any more complicated treatment. So nothing really new … nothing different there*.’ (D01: Dentist, Scotland)

As with the other approaches, preventive treatment also has a meaning of being ‘conventional’, in the sense of being routine. However, for some, participating in FiCTION involved giving more advice than usual:

‘*I do find that outside of FiCTION, we try to deliver some of that advice. But there's a lot of advice to deliver there … I feel that sort of I can give that time to my FiCTION patients. But outside of FiCTION, they get some of that advice, but perhaps not as much as I'd like*.’ (N01: Dentist, North East)

This may indicate that outside the FiCTION RCT, performing the entity of providing preventive advice is affected by the underlying infrastructure of NHS dentistry (as this dentist suggests).

Nevertheless, while prevention is something ‘*we do all the time*’ (D04: Dentist, Scotland), several participants suggested that the PA arm was insufficient as a way of managing children with carious lesions:

‘*My least acceptable would be to do nothing really. I mean, I do do nothing in some cases, you know, if there are large cavities, which is self-cleansing. But a number of patients I have treated and done nothing under the trial, I felt would have been better treated the way I normally treat*.’ (N06: Dentist, North East)

*‘I think we’d get accused of leaving cavities to progress if we went on the prevention arm*.’ (S06: Dentist, Yorkshire)

The idea of prevention alone as ‘doing nothing’ was a shared meaning, and DPs explained that parents could be unsatisfied and that other organisations could accuse them of ‘*supervised neglect*’ (S05: Dentist, Yorkshire).

Participants questioned the effectiveness of preventive treatments, such as applying fluoride varnish or providing advice on oral care and diet, which were not seen as ‘interventions’ in the same way as fillings or PMCs were. The inclusion of the PA arm in the FiCTION RCT frames this as an approach that may be as effective as the other treatment arms. Some DPs acknowledged successful outcomes on the PA arm. However, the language used in most interviews indicated that DPs believed the PA arm might not be an effective way of managing children with carious lesions, particularly if patients and parents did not follow advice. Other participants described how they were worried about the dental caries ‘getting worse’ due to the provision of preventive treatment alone.

‘*My theory is if you did prevention alone, it's up to the parents and the children then. It's in their hands. And what we see walking through the door is even though we do prevention most of the time, it's not working.*’ (L02: Dental Nurse, Yorkshire)

While DPs recognised the relevance of socioeconomic factors, their responses highlight that providing preventive treatment involves competences on the part of the patient and their parents, as well as the DP. The practice of providing best practice prevention is connected to oral hygiene behaviours in the home such as tooth brushing, which can also be understood as social practices (involving the materials of a toothbrush and toothpaste, the meaning of achieving oral hygiene and the competences of understanding why brushing is necessary and the skill to brush in an effective way). These data indicate that DPs were often not confident that patients were performing the relevant practices frequently and competently enough. This reliance on positive parent and child behaviour made prevention alone difficult to perform. There is the possibility that the entity of providing prevention alone may change over time and take on the meaning of being an effective treatment. However, these links were generally not being made by DPs in this research, because of the connection of this practice to patients’ daily oral hygiene behaviours being less than optimal.

### Deviation from allocated arm

DPs were asked about deviating from the FiCTION clinical protocol. Some reported deviating from the protocol in order to act in the best interests of the patient:

‘*I can think of one case I've done where I varied from a conventional arm to putting on a Hall crown, just because I felt the child would manage better.*’ (N10: Dentist, North East)

‘*I've had a couple of … I think people in the preventive arm who've had to get some wee fillings. [ … ] It has been my decision because I don’t want to leave a child in pain. You know, if somebody comes in in pain, you have to do something*.’ (D02: Dentist, Scotland)

Other participants demonstrated discomfort that they were not able to do what they felt was ‘best’ for a patient due to the random allocation of treatment arms:

‘*So I wasn’t not happy about doing it but wondering whether or not you were doing the best for that child in that instance, I suppose’* (D03: Dentist, Scotland)

‘*It's been a little bit challenging because probably some of the patients that we would have done in different arms as to the way to treat them, it was already picked for you*.’ (L02: Dental Nurse, Yorkshire)

Managing children with carious lesions during routine dental care involves the DP selecting the best treatment option. In the context of the FiCTION trial, being told what to do in each case could result in a DP feeling ‘guilty’ or finding it ‘difficult’ not to intervene. In order to better understand these deviations and reported feelings of guilt, it is necessary to consider how participants spoke more generally about managing children with carious lesions.

### Managing children with carious lesions

DPs spoke about the three treatment arms in the context of the wider ‘bundle’ of social practices which we refer to as ‘managing children with carious lesions’ [15]. Although DPs and parents may have referred to this in different ways, this phrasing emphasises that treating carious lesions is patient-centred. Managing children with carious lesions emerged from the data as a recognisable ‘bundle’ of social practices that describes one aspect of what DPs do, both as part of the FiCTION trial and in their everyday work. The way in which DPs spoke about managing children with carious lesions reflected ideas of what ‘doing dentistry’ *should be*.

#### Selecting a suitable treatment option

Managing children with carious lesions involved selecting a suitable treatment option for each particular patient. This revealed that treatments are themselves embedded within wider social relationships, as DPs are recruiting existing patients:

‘*There's no point in having a fancy plan about what you're going to do with a child who can't keep their mouth open or a child who’s frightened of local … So very much the child will dictate what your treatment plan is*.’ (D01: Dentist, Scotland)

Here treatment options are contingent on the child being able to perform as a patient who may or may not co-operate with certain treatments. DPs therefore had an interpersonal knowledge about the child, developed through a relationship over time. In some cases, this relationship clearly impacted on their willingness to deliver treatment from the limited range of options available within the arm to which the child had been randomised.

In addition to this, the lack of definitive evidence on the most effective way to treat dental carious lesions can also make it difficult for DPs to select the best treatment option in their day-to-day work:

‘*Well, you don’t know what’s best. And I think dentists are scared of saying that because then they think that, “Well, I’m looking as though I’m a bit thick and I don’t know what the treatment is,” you know. “Or I’ve been providing you with this, and now I’m telling you I don’t know, you know, I don’t know what’s best.”*’ (S07: Dentist, Yorkshire)

The competence of being able to select the ‘best’ treatment for each patient requires practical know-how in the sense of which entity is best under which circumstances for a particular child. It also reveals the pressure to maintain a sense of professional confidence in front of patients.

#### Obtaining parental trust

Additionally, participants referred to parents wanting and trusting DPs to decide what was best for the child concerned:

‘*We had to obviously give a bit of information on how the three different arms [worked]. They then just wanted to go with what I thought was the best*.’ (E01: Dentist, Scotland)

This was understood as the parents ‘trusting’ the DP (as long as the way in which DPs performed the activity of managing children with carious lesions reflected ‘clinical evidence’):

‘*They really trust us to be honest with you, because, I mean, whatever we say, almost goes so if we say, “[Name] we’re going to try this on you,” and there is some clinical evidence that it does work, they’re happy to go with it*.’ (S07: Dentist, Yorkshire)

Parents therefore recognised that DPs were engaged in the activity of managing children with disease, which involved displaying the competence of being able to select the most appropriate treatment, and which meant they were acting in the best interests of the patient.

Within the FiCTION RCT, the random allocation sometimes interfered with these relationships and undermined this competence. DPs found that parents questioned the effectiveness of a prevention-alone approach:

‘*When they have this prevention only, they’re a bit like cautious and a bit like not sure whether it would work*.’ (LDN01: Dentist, South East)

This reflected an expectation the DP should ‘do something’:

‘*There's probably an expectation on the parents that some sort of active treatment is provided for the patient, some sort of restorative treatment*.’ (N01: Dentist, North East)

Furthermore, according to DPs, the prospect of randomisation to the PA arm did cause parents to decline to participate in FiCTION due to the perceived lack of intervention:

‘*I did have some parents decline going on the trial because they didn’t like the idea that I wasn’t doing anything if they went on the prevention arm even though, we were doing something, they just perceived it as I wasn’t doing anything.*’ (D04: Dentist, Scotland).

The DPs suggested that parents were more willing for their child to have an unfamiliar treatment (in this trial, the PMC) if it was explained:

‘*I think if you try and explain the benefits of the crown and…I guess it's how you spin it a little bit, how you sell it to them, how the uptake is going to be. And I guess if you try to sell it that this is going to be the best for their child, most people have been fine in the end*.’ (N01: Dentist, North East)

The language used here, around ‘explaining’ and ‘selling’ an option, points to the influence DPs have in shaping how parents interpret a particular treatment. DPs convey the meanings associated with different entities when they explain treatment options. These meanings develop through the repeated performance of particular entities. Thus the material (the PMC) could become associated with a meaning (being ‘effective’) as this social practice evolves over time. Nevertheless, parents can help to establish practices within dentistry by accepting particular meanings, materials and competences, or they can contest practices by questioning what they are told by DPs. In these accounts, the materials involved in the C + P and B + P arms are recognised by parents as acceptable restorative treatments, while ‘just keeping teeth clean’ is viewed as ‘insufficient’.

### Future management of children with carious lesions

DPs spoke about how participating in the trial might shape their future management of children with carious lesions in routine clinical dental care.

#### Treatments outside the FiCTION trial

DPs described preferences for particular future treatment options outside the FiCTION trial. For example, some DPs spoke about preferring to avoid conventional methods in the future:

‘*I think I would personally, you know, if … if given the choice, most of the time I'd try and avoid the conventional methods*.’ (E01: Dentist, Scotland)

Although participants spoke about moving away from delivering LA, removing carious tissue and filling teeth, it was recognised that the meaning of ‘drilling and filling’ as being ‘conventional’ could make this transition difficult:

‘*I’m still filling children’s teeth. That’s what I was trained to do and it’s very hard to get out of a rut isn’t it?*’ (D04: Dentist, Scotland)

That other DPs who are ‘doing conventional all the time’ and might find the performing the activity of sealing carious tissue into teeth ‘*more of a challenge*’ (D03: Dentist, Scotland) was also identified as a potential issue. The meaning of this activity as being ‘expected’ to some parents and children can also reinforce its meaning as being ‘routine’.

However, DPs were willing to move towards biologically-based treatments, rather than removing carious tooth tissue and providing fillings:

‘*I don't feel I'm cheating now if I put on a PMC. You know, before I would have tended to think well, in most cases you should either be doing an extraction or you should be doing a conventional filling. So I think … I think I probably will feel less guilty about doing a PMC.*’ (D01: Dentist, Scotland)

The idea that performing a particular entity is ‘cheating’ reflects that this is not associated with the meaning of doing what one is ‘supposed to’; sealing-in carious tooth tissue is not an established social practice. However, the interviews demonstrated that DPs were generally positive about non-conventional options if they had been trained in these, understood them to be ‘effective’, and were able to display the relevant competences.

Nevertheless, when DPs did not feel confident about the techniques involved, their responses suggested that the ‘unfamiliarity’ of the activity of sealing-in carious tissue could discourage them from engaging in this way of managing children with carious lesions in their primary teeth:

‘*The biological way is probably the best but it’s deciding when something is bad enough to need a Hall crown or whether you could just make it self-cleansing and put in some glass ionomer or something like that. That’s the difficult call to make*.’ (G01: Dentist, Scotland)

A central part of these judgements was a DP’s own judgement about their ability to perform a particular entity. Entities can be performed by people with more or less experience, and this can impact on the outcome of a particular performance. In order to maintain a practice over time, people who perform a particular entity need to familiar with the materials and develop the required competences. For example, the interviews suggested that training in the placement of Hall crowns might be helpful, particularly for clinicians unfamiliar with the technique. There was also a need expressed to display confidence in the techniques required within the biological arm.

Other DPs spoke about their growing awareness of the importance of prevention:

‘*I think really concentrating on the different advice so I can avoid having to do too many clinical interventions on children. Definitely the way to go.*’ (E01: Dentist, Scotland)

Nevertheless, providing prevention alone was still seen as being a potentially ‘insufficient’ way of doing dentistry:

‘*I don’t like the prevention arm. I feel like I’m …* ’ (S05: Dentist, Yorkshire)

‘*I feel I’m not doing my job*.’ (S06: Dentist, Yorkshire)

‘*Yeah, yeah*.’ (S05: Dentist, Yorkshire),

(Exchange from group interview, Yorkshire)

Some DPs planned to combine prevention with biological treatment options:

‘*I would like to think of more, far more prevention, and a bit more biological would be the future*.’ (N07: Dentist, North East)

This data shows that the entities of drilling and filling teeth, sealing-in carious tooth tissue and providing prevention alone carry meaning for DPs, patients and their parents. But more than this, these meanings are themselves embedded in a complex set of relationships. The meaning of any entity reflects the experience of patients and their parents and new evidence produced from research, as well as meanings for DPs (see Table 2). As a consequence, the meaning of aspects of ‘doing dentistry’ can change as new entities emerge and are performed. Through training, DPs subsequently develop new competences with different materials, such as the understanding of how PMCs work and the skill to apply them. Through repeated performance, this entity of sealing-in carious tooth tissue develops the meaning of an effective treatment, and this is conveyed to patients and parents.

#### Accepting the results of the FiCTION trial

When asked a hypothetical question about what the results of the RCT might show (given the results were not available at the time of the interviews), DPs spoke about being willing to accept the results and potentially change how they treated patients:

‘*I will happily do what … if it is shown that doing that makes a significant difference to the lives of the patients that we look after, happily. It's my job to look after them with the best knowledge that I have*.’ (S02: Dental Nurse, Yorkshire)

‘*I'd be really interested to find out what, you know, what the results are. And absolutely I will change my practice if … if there's something that's massively better, then, yeah*.’ (D02: Dentist, Scotland)

This understanding of what it means to be a DP was reflected in a number of interviews. Participants recognised a responsibility to practice evidence-based dentistry, by learning from research.

In their interviews, participants spoke about the value of evidence, and their responses indicate that a trial such as FiCTION can alter the meanings of particular entities and thus how these are performed going forward. Nevertheless, it is important to recognise that experiential knowledge gained outside of FiCTION was also valued:

‘*But that's when people start to accept things, because it's not just the … it is important to get evidence, but it's the word of mouth of people's experiences.*’ (N11: Dental Therapist, North East)

Participants’ valued their own experiential knowledge, and that of colleagues, passed on by ‘word of mouth’.

The interviews therefore highlight that practical skills involved in managing children with carious lesions are gained through experience and discussion with colleagues, as well as through engaging with research:

‘*The whole purpose of evidence-based, in my understanding, is that it’s not just based on scientific research, it’s in consultation with the patient and clinical expertise as well. So, I think, you know, combining more together, you’ll get an individual plan for the patient provided you can justify what you’re doing. I think that’s when clinicians will feel confident, you know, about the care that they provide*.’ (S07: Dentist, Yorkshire)

Other DPs suggested the results of the trial would not be definitive, and thus it would be important to continue to draw on experiential and interpersonal knowledge in order to manage children with carious lesions and work in the best interests of the patient:

‘*But I don't think we’ll get anything as definitive as that so we will carry on I would think looking at each child individually [ … ] And giving treatment that best works for them.*’ (D01: Dentist, Scotland)

In relation to this, DPs also expressed the view that it was important to have a choice, allowing them to demonstrate the skill of selecting the right treatment option for a particular patient:

‘*I still think you need to give clinicians a choice. I don’t think … even if the trial came back and said “right, that’s what we need to do, all the time, every time”. There are situations where you may not be able to do it and you need the other options*.’ (D04: Dentist, Scotland)

Participants suggested that they wanted to manage children with carious lesions in a way consistent with the meanings, materials and competences of ‘doing dentistry’.

## Discussion

This qualitative study with DPs illustrated how managing carious lesions in the primary teeth of young children was a recognised bundle of social practices involving a range of options. For each individual child, this involved the skill of selecting the most appropriate treatment option. This could be understood as connected to ‘doing dentistry’, a social practice involving materials (dental training, General Dental Council standards, published research), other competences (including the skills of explaining one’s approach to patients and parents, achieving trust and displaying professional confidence, as well as understanding of research) and meanings (acting in the best interests of the patient, DP as trusted, evidence-based dentistry). ‘Doing dentistry’ involves drawing on experiential and interpersonal knowledge, as well as research-based evidence, in order to act in the best interests of one’s patient as an individual. Participants indicated that parents/guardians shared this understanding, trusting DPs to treat each child in the ‘best’ way possible.

This understanding of ‘doing dentistry’ and the bundle of social practices involved in managing children with carious lesions meant that some DPs, when providing treatment as part of FiCTION, deviated from the allocated trial arm in order to treat a particular patient in a different way. In these cases, DPs referred to practical know-how, considering how well a child would ‘cope’ with the treatment and assessing the extent of the carious lesions, as well as referring to their own experiences of using different treatment options. Such treatment deviations indicate the importance of being able to select the most appropriate treatment option; when DPs continued with a treatment they felt was less than ideal, they described this as a ‘difficult’ and ‘uncomfortable’ experience. Being subject to the constraints of an RCT highlights how treating children within FiCTION contrasted with day-to-day social practices of doing dentistry.

A key principle within RCTs is that of equipoise, that is, uncertainty about whether one treatment will be beneficial over another [32]. There is a distinction between individual equipoise (the view of one clinician) and community equipoise (also referred to as collective or clinical equipoise, which is the collective uncertainty of the community) [32]. Equipoise can change over time as new evidence emerges. When clinicians are not in individual equipoise, they describe feeling ‘discomfort’ and may not recruit patients or may express a treatment preference [12, 33]. As equipoise can change over the course of a trial, DPs in this study may not have expressed preferences earlier in the process. Although equipoise is a requirement of RCTs, previous research suggests that this is not fully understood by the general population [34]. From the DPs’ perspectives, admitting to uncertainty undermined parents’ trust as they did not appear to be engaging in the recognised activity of managing carious lesions. Again, not being able to ‘do dentistry’ in the familiar way (i.e. explaining one’s choice of treatment to patients and parents) can be an uncomfortable experience for DPs.

DPs engaged in three social practices as part of FiCTION in order to treat carious lesions: ‘drilling and filling’ with prevention, sealing-in carious tooth tissue with prevention and providing best practice prevention alone. Each practice includes an entity comprised of different elements, which we have classified as materials, meanings and competences according to SPT [14]. These entities are repeatedly performed by DPs which reinforces the links between particular materials, meanings and competences. The idea of social practice captures both the entities (the recognisable ideas that can be spoken about) and the performances (doing an activity). Performing these entities takes place both within the RCT and outside it. The social practices that were part of this trial are also part of the day-to-day work of dentistry, and are understood in terms of being a DP. The component elements, and the relations between them, develop outside of the context of the trial. DPs therefore had particular understandings of the treatment arms which influenced whether they were prepared to follow the clinical protocol for each child in all instances. For example, several identified the PA arm as ‘insufficient’ on the basis of knowledge gained prior to commencing the trial. As a result, they chose to deviate from the clinical protocol in order to treat their patients in a different way.

According to the uncertainty principle that governs RCTs, healthcare professionals who take part should be unsure as to which option, or trial arm is superior [32]. Therefore, if these professionals are in individual equipoise, each constituent entity should have the meaning of being an ‘acceptable’ option for all patients. However, these new connections cannot be forced; connections between elements occur through dynamic processes of association as entities are repeatedly performed, and cannot be controlled by one person or group (in this case a research team) [14]. Several participants indicated that they felt that the social practice of providing prevention alone was insufficient without further intervention. It may be possible that this social practice is still more of a proto-practice; as such its meaning as an effective treatment for carious lesions is not particularly well established in the context of oral care. Yet other participants who did follow the clinical protocol were surprised at the success of using prevention alone to manage carious lesions. By situating the social practices involved in FiCTION in the wider context of ‘doing dentistry’, we can see that some DPs struggled with equipoise [33]. As has previously been identified, involvement in RCTs can be an intellectual and emotional challenge for healthcare professionals [33]. If a DP is not in individual equipoise, treating patients according to a randomly selected treatment arm may feel uncomfortable. During their training to take part in RCTs DPs need to be prepared for these challenges.

The treatment options involved in this trial were social practices that already carry particular meanings for DPs, meanings which connect and disconnect from existing practices over time. These processes of connection and disconnection occur as DPs repeatedly perform different entities, and experience treatments as successful or not. DPs also learn from verbal and written accounts of other people’s performances (for example, through conversations with colleagues or published research), which can help to strengthen the links between particular elements. As a result, the social practices themselves are subject to instability and change with some being better established than others. This research found that repeatedly performing new entities over time can contribute to the formation and deformation of links, as with the Hall technique (which had started to carry the meaning of being ‘effective’ rather than ‘unfamiliar’ for participants in this study). However, individual DPs need to be introduced to the social practice of sealing-in carious tooth tissue through gaining familiarity with the relevant materials, training in selecting and fitting crowns and reading research that demonstrates the effectiveness of this approach.

Participants were also questioned as to how they anticipated managing children with carious lesions in the future. The aim of FiCTION was to compare the clinical and cost-effectiveness of three treatment strategies. DPs acknowledged the value of evidence and stated that they would use the results of FiCTION to inform how they practiced dentistry. However, several rejected the idea that FiCTION would identify one option that would be most effective in every situation. Participants emphasised that they expected to continue to display practical know-how; using existing experiential and interpersonal knowledge to select the best option for each individual child. Our research shows that meanings of effectiveness are strongly linked to particular materials and competences, and these connections may be difficult to break, despite the possibility of different ways of managing children with carious lesions. Recruiting individual DPs to new social practices is likely to be a gradual process and therefore challenging to implement into a trial without a longer lead-in time. Following the FiCTION Trial, further work, using Normalisation Process Theory, could investigate the implementation of interventions to manage children with caries in general dental practice [35, 36].

### Strengths and limitations of the study

Many studies exploring the attitudes and beliefs relating to dental care rely upon participants considering how they might feel in an abstract way, imagining how they would be react or behave in a particular instance. Participants in this study however have actively been engaged in all of the caries management strategies discussed and thus interviews reflected on attitudes and beliefs based up real life experiences. The findings of this study should be viewed in light of the study limitations. The sample size of 31 is consistent with qualitative research, and is not a limitation. Nevertheless, due to the small number of participants involved, it was not possible to draw conclusions about the differences between individual dentists as compared to those between dentists (as a group), dental nurses and dental therapists.

The DPs were interviewed on one occasion (towards the end of the trial, but before the findings were available). A longitudinal study design, involving repeated interviews, may have shown how views changed over time. This may be a useful approach in future qualitative research evaluating clinical trials.

### Reflexivity

This component of the study was designed by the trial management group with further support from two sociologists in the use of SPT. The decision to use SPT to guide the analysis was driven by the data itself. While a full range of DPs were interviewed it was not possible to interview those who had withdrawn from the study.

The interviews were conducted by four experienced qualitative researchers, two of whom were clinical dental academics, to enable the more technical aspects of the dental treatment to be explored while also ensuring the broader socio-cultural and economic aspects were not ignored. The analysis was conducted independently, by two members of the team initially, with ongoing discussions with the co-authors. The qualitative data analysis was conducted before the results of the analysis of the primary and secondary outcomes were revealed to the researchers involved.

## Conclusion

DPs describe engaging in the various social practices that were bundled together as ‘managing children with carious lesions’, both within the FiCTION RCT and on a day-to-day basis. This involves selecting what the DP views as the most appropriate treatment option for an individual patient. DPs demonstrate practical know-how in order to do this.

According to DPs, this bundle of practices is also recognised by parents, who trust the practitioners to act in this way as part of ‘doing dentistry’. Treating patients according to a randomised option undermines this trust and what it means to be a DP (i.e. what it is perceived that DPs *should* do). As a result, DPs may struggle to comply with randomly allocated treatments in a RCT setting and would benefit from further training in evidence-based research principles.

Future trials may benefit from considering how professional involvement in a clinical trial fits with existing day-to-day practices. Further work to prepare healthcare professionals for the intellectual and emotional challenges of trial participation may be beneficial.

## Supplementary information

**Additional file 1.** Interview and Focus Group Topic Guide.

## Data Availability

The datasets analysed in the current study are available from the corresponding author on reasonable request.

## Abbreviations

B + P: Biological management of carious lesions with best practice prevention
C + P: Conventional management of carious lesions with best practice prevention
DP: Dental professional
FiCTION: Filling Children’s Teeth: Indicated Or Not
GDP: General dental practitioner
LA: Local anaesthetic
PA: Best practice prevention alone
PMC: Preformed metal crown
RCT: Randomised controlled trial
SPT: Social practice theory

## Footnotes

1 The dentinal lesion was either cavitated or non-cavitated and was diagnosed by visual dental examination alone or with bitewing radiographs.

2 For example, the entity of drilling and filling a tooth comprises **materials** such as the filling material, the drill and the carious lesion in a tooth; **competences** refer to understanding why fillings work, as well as knowing how to drill away carious tooth tissue and apply the filling material. ‘Drilling and filling’ involves **the interaction** of these particular materials and the competences used and various **meanings**, such as ‘restoring the tooth’, as well as ‘effectively treating carious tissue.’

3 DPs actively drilling and filling teeth (performing the practice) sustain the links between elements within the entity (a particular set of materials, competences and meanings). Thus as a practice, ‘drilling and filling’ is recognisable to successive generations of DPs and patients, both as an idea that can be talked about and taught in dental schools, and an activity that DPs perform in their day-to-day work of ‘doing dentistry.’

4 Drilling and filling as a practice has changed over time, for example as composite emerged as an alternative filling material to amalgam, and air turbine drills require different skills to older models. New elements have the potential to become linked to existing meanings (such as ‘restoring the tooth’ and ‘effectively treating carious lesions’), and disconnect from older ones (e.g. drilling as ‘laborious’).
