# Supplementary material for: Dental professionals’ experiences of managing children with carious lesions in their primary teeth – a qualitative study within the FiCTION randomised controlled trial
Source: BMC Oral Health. 2020 Mar 4;20:64. doi: 10.1186/s12903-020-1051-7 (PMC7057668; doi:10.1186/s12903-020-1051-7)
Supplement: Supplementary file 1 — Additional file 1. Interview and Focus Group Topic Guide. [file 12903_2020_1051_MOESM1_ESM.docx]

**Additional file 1: Interview and Focus Group Topic Guide**

*Flexibility should be used when undertaking the interviews and applying the topic guide in terms of wording of questions, order of questions, use of probes/prompts and every opportunity made to allow participants to raise their own issues.*

**Opening:**

- Write names on stickers
- Thank you for participating
- Purpose of focus group: Part of the National Institute for Health Research-funded FiCTION trial that your practice is taking part in. As you are aware, the aim of the FiCTION trial is to compare the clinical and cost-effectiveness of three treatment strategies for dental caries in children. In this part of the FiCTION trial we want to find out what you really think about these different strategies and how well they work (or not) in your dental practices.
- Few things to run through before we start
- Confidentiality – information collected during the study is confidential and access will be restricted to our research team. When we analyse the data we won’t use your real name but we will use the information you provide on the participant questionnaire (e.g. your job role). Some of your comments may be included in a report on the study or in articles for scientific journals but these will be completely anonymous. Please don’t repeat what other people say outside of this session
- No right or wrong answers
- Everyone’s views are of interest
- Aim to hear as many different thoughts as possible
- Likely to be different views, feel free to say what you think – OK to agree/disagree with others
- Don’t wait to be invited before stepping in, but don’t talk over each other
- Need to record so we can remember what is being said
- Might make some notes while you’re speaking – just to jog memory
- Any questions?
- Consent form

**TURN ON AUDIO RECORDER**

**Introductions:**

- Can you introduce yourself and tell us a bit about your background (e.g. age when qualified, years in practice, further qualifications, full-time/part-time, work in other clinical environments [hospital/teaching], children as a % of caseload) *(focus group moderator and note-taker begin)*

**Perspectives on patient and parent/guardian preferences:**

- What expectations do you think children have about how their dental caries will be managed?
- What expectations do you think parents/guardians have about how their children’s dental caries will be managed?
- Do these expectations cause you to feel pressure to manage the children’s dental caries in a particular way? Why/why not?
- Have you deviated from the allocated treatment arm for any patients because of this? If yes – why?
- How do patients respond to each of the three treatments?
- Which strategy do you think is most acceptable to them? Why?
- Which do you think is least acceptable? Why?
- How do parents/guardians respond when told what treatment their child would receive?
- Which strategy do you think is most acceptable to them? Why?
- Which do you think is least acceptable? Why?
- Why do you think some participants have dropped out?
- In your experience do any of the treatment strategies require more patient management than the others for adequate compliance?
- What do you think could be done to improve the acceptability of the management of dental caries for children?

*Probe: each specific strategy*

- What do you think could be done to improve the acceptability of the management of dental caries for the parents/guardians of the children?

*Probe: each specific strategy*

**Previous experience:**

As you know, the three treatment strategies we’re comparing are conventional management + best practice prevention, biological management + best practice prevention and best practice prevention alone.

- What did you know about each of the three management strategies before taking part in the FiCTION trial?
- What training had you received in delivering the three management strategies before taking part in the trial?
- What experience did you have of delivering the three management strategies before taking part in the trial?
- What previous experience did you have of treating children?
- What previous experience did you have of managing children’s behaviour?
- How have you found delivering the management strategies that you hadn’t delivered before?

**Experience of providing the three strategies in the trial:**

- How was each of the management strategies carried out in your practice?

*Probe: what was delivered?, who delivered it, what did they do, how did they do it?*

- How have you found following part 1 of the trial protocol: ‘Participant allocation and treatment planning; which treatments for which study arm’? Why?
- How have you found following part 2 of the trial protocol: ‘Supporting information for protocols; how to carry out designated treatments’ for each of the three management strategies? Why?

*Probe: time to do so*

- How do you find taking radiographs? Why?
- How do you find giving local anaesthetics? Why?
- Are you confident in your ability to deliver each of the three management strategies? Why/why not?
- Are you confident in delivering local anaesthetic? Why/why not?
- Are you confident in delivering pulp therapy for primary teeth? Why/why not?
- Are you confident in the long-term monitoring of teeth with Hall Technique crowns? Why/why not?
- Are you confident in your ability to carry out prevention that will arrest decay? Why/why not?
- Are you confident in your ability to carry out prevention that will lead to behaviour change in parents/guardians and children? Why/why not?
- Which management strategy is the most difficult to deliver? Why?
- Have you ever deviated from the protocol? If yes – what are the reasons for this?
- Do you have the resources you need to deliver each of the three management strategies? If no – what do you need that you don’t have, e.g. equipment, time, staff?
- Thinking about the dental contract arrangements you work under, either the practice contracts or your own contracts, how do you feel about each of the three management strategies?
- Have there been changes in your practice, e.g. new owners (corporate bodies)?
- What are your views on whether each of these three management strategies benefits children’s dental health?

*Probe: Do you believe that they achieve the goal equally well? Why/why not?*

- What are your views on whether each of the three management strategies is what the dentist and/or other dental team members should be doing as part of their job role?
- Do you have any worries or concerns about managing the children’s dental caries with any of the three management strategies? If yes – what are they?
- How well has your dental team as a whole adapted to the changes required to deliver the three strategies?

*Probe: using new materials, ICDAS caries coding, remuneration*

- With a free choice, which of the three management strategies would you choose to deliver? Why?
- Would this differ between situations? What influences your decisions?

**Future management of dental caries:**

- How do you currently manage children who are not part of the FiCTION trial? Why?
- How will you manage children who have taken part in the trial once the trial is over? Why?
- How will you manage other children who have not taken part in the trial once the trial is over? Why?

*If they would manage them in the same way that they did prior to participating in the trial:*

- Why? What would have to change for you to manage them differently?

*If they would manage them differently from how they would have prior to participating in the trial:*

- Why? How will you implement the change?
- Does your dental practice/practice owner allow changes to be made to how you treat dental diseases as new technologies or evidence emerges?
- Do the General Dental Council (GDC), your defence society and your NHS Board allow changes to be made to how you treat dental diseases as new technologies or evidence emerges?
- Will the cost of resources for particular management strategies influence your decision about how to manage dental caries in children in the future? Why/why not?
- If the results of the FiCTION trial show that the outcome for best management requires a change in how you manage dental caries in children, what will you do?

*If they would not implement the strategy for best management:*

- Why not?

*If they would implement the strategy for best management:*

- How would you find making that change?

*Probe: would habits, preferences, past experience make it difficult?*

Translating the findings of research into clinical practice requires practitioners firstly to be aware of the findings, secondly to accept the findings and finally to adopt the findings into their practice.

- How can we best promote awareness of the findings of the trial among dentists and dental practice staff?
- How can we best encourage acceptance of the findings of the trial?
- How can we best encourage adoption of the treatment strategy that the trial finds to be the best way to manage dental caries in children?

**Training needs:**

- What are the skills required to deliver each of the three management strategies?
- Do you have all of these skills within your team? If no – what don’t you have?
- How do you tell the patients and parents what you are going to do for each of the three management strategies?
- Were you taught communication strategies, in particular strategies for communicating with children, in your undergraduate or postgraduate training?
- How useful was the training for the FiCTION trial in equipping you to deliver the protocols for each of the management strategies?

*Probe: usefulness of clinical skills labs, lecture-based training, in-practice training*

- Were you trained in how to communicate what you were going to do to the patients/parents?
- How well prepared did you feel for taking part in the trial following the training you were given?

*Probe: prepared for: administration, delivering the strategies, managing children’s behaviour*

- How do you think the training could be improved?

*Probe: type of training, materials, number and length of sessions, practice*

**Experience of being involved in research:**

- What do you like about being part of the FiCTION trial? Why?
- What do you not like so much? Why not?
- Have you encountered any difficulties running the trial in your practice?

*Probe: problems with recruitment, consent, paperwork, outcome measurement (e.g. ICDAS), time to manage the trial commitment, radiographs*

- What have you learned from being part of the FiCTION trial?
- How have you found the communication between the research team and the practice?

*Probe: how well does the research team communicate with you?, do you like the emails, face-to-face meetings, newsletters?, how well can you communicate with the research team?*

- What is your experience of keeping participants in the trial?
- Why do you think some participants have dropped out?
- How do you think being involved in a trial has changed the patients’ approaches to treatment?
- Would you be willing to be involved in similar trials in the future? Why/why not?

**End:**

- Is there anything you would like to add, anything we’ve missed out?
- Is there anything else the note-taker would like to ask about?
- Thank you for participating – it’s been very helpful and will help us to make recommendations to improve the future management of dental caries in children.
